# Opportunities and Challenges for Regional Coordination of Infectious Disease Control: Comment on "Operationalising Regional Cooperation for Infectious Disease Control: A Scoping Review of Regional Disease Control Bodies and Networks"

**DOI:** 10.34172/ijhpm.2022.7533

**Published:** 2022-12-10

**Authors:** Claire J. Standley, Erin M. Sorrell

**Affiliations:** ^1^Department of Microbiology & Immunology, Center for Global Health Science and Security, Georgetown University Medical Center, Washington, DC, USA; ^2^Heidelberg Institute of Global Health, University of Heidelberg, Heidelberg, Germany

**Keywords:** Disease Control, Infectious Disease Networks, Scoping Review

## Abstract

This commentary cites a scoping review by Durrance-Bagale et al (2021) on how regional bodies have approached infectious disease control to determine if those lessons could be applied to assist the Association of Southeast Asian Nations (ASEAN). The author’s work is timely and highlights the importance of recognizing and understanding regional context, governance and operational structures to then design effective regional networks. Most factors highlighted as enablers and constraints are quite expected, including stakeholder mapping, a clear mission space with goals and objectives, outreach and advocacy to receive buy-in, political will and sustainable funding. We suggest below that there is an opportunity for further systematic and operational research of enablers and constraints for regional infectious disease control bodies, one that expands on infectious disease control while also continuing to take into account governance, legislative and organizational factors, and strongly emphasizes the development and application of clear metrics to create better measures of impact.

 Infectious disease networks established to build capacity and encourage collaboration between national and regional stakeholders are essential for both national and regional health systems strengthening. These networks can be leveraged to connect global policies to national priorities, strengthen capacities for the prevention, detection, response and recovery to disease threats and even organize the procurement of equipment, countermeasures and consumables on behalf of its members. Cooperation can range from informal one-off approaches to more formal systematic processes of policy development and implementation through regional networks and organizations. For example, the Connecting Organisations for Regional Disease Surveillance (CORDS) network supports 6 member networks, in 28 countries in Africa, Asia, the Middle East and Europe. Each network promotes regional collaboration for infectious disease detection and control.^1^ Laboratory networks, such Foundation Mérieux’s West African Network of Biomedical Analysis Laboratories^2^ works to promote and strengthen laboratory systems across seven West African countries through regional and cross-border approaches. These organizations not only assist in communication and coordination across its members but also act as amplifiers of regional capacities and policies to international organizations as well as serve as intermediaries interpreting and adapting global agreements and policies to its member states’ national context and priorities. Unfortunately, the coronavirus disease 2019 (COVID-19) pandemic has shown an inward facing approach to outbreak management and response. This scoping review by Durrance-Bagale et al^3^ sought to examine how regional bodies have approached infectious disease control, and see if those lessons could be applied to assist the Association of Southeast Asian Nations (ASEAN) Center for Public Health Emergency and Emerging Diseases.

 Most factors highlighted as enablers and constraints for regional networks are quite expected, including stakeholder mapping, a clear mission space with goals and objectives, outreach and advocacy to receive buy-in, political will and sustainable funding. Enhanced disease surveillance and field epidemiology training were highlighted as two of the main technical areas of regional cooperation related to infectious disease control. Interestingly, laboratory capacity was highlighted as an enabling factor, rather than the direct target of regional cooperation. The regional examples identified in the paper were drawn from all continents; most were aligned with existing political or administrative regional networks, and had formal status among member states in that sense. Some represented more organic, bottom-up collaboration between geographically proximate countries, for example the Middle East Consortium on Infectious Disease Surveillance, a CORDS member.^4^ While the examples provided by the review represented one or more key enablers for disease networks the authors did not take into consideration regional networks established by an external entity or donor. This decision may have been due to the scoping focus on how best to inform ASEAN’s regional disease control center, which has faced its own challenges.^5^ There are however some advantages in considering external entities. Review and analysis of networks like the US Centers for Disease Control Disease Detection centers can provide roadmaps on how best to integrate donor/external interests alongside national priorities when it comes to disease control.^6^

 An interesting observation cited by the authors was where heterogeneity of countries was as an obstacle. This could be a useful measure for future analyses challenging the concept of using regions as a parameter for comparison and/or analysis. Members within regions can be quite different in political, governance, societal and religious systems while others are more homogenous. The obstacles faced by one network in agreeing to a common language in developing terms of reference and an operating and/or governance structure can be quite different from another. Take for example the comparison of a regional network of Northern European countries and one established across the Horn of Africa. This paper notes that enablers and constraints were consistent across regions however, without providing specific examples of how these factors demonstrated similar across regions, it is challenging to derive actionable lessons learned that could be used in the development or establishment of future networks. To this end, we suggest that an important next step to build on broad level overviews, such as this scoping review, could be the development and application of detailed methodologies for effective network building. Such tools can help to establish an area of practice that can then be applied in the development and/or revision of regional networks.

 Conflict and political instability were highlighted as some of the top challenges to establishing regional collaborations. One must consider building regional capacities from subnational networks, that is surveillance, laboratory or other infectious disease-centered technical units that can survive political turmoil, insecurity and/or conflict in a country or across a region. The Middle East Consortium on Infectious Disease Surveillance is an excellent example of how regional networks can be established to circumvent geopolitical challenges.^7,8^ This network established by public health experts and ministry officials from Israel, the Palestinian Authority, and Jordan enhances national laboratory-based surveillance networks. It was founded with a priority towards detection and response to foodborne diseases but has expanded to include respiratory and vector borne diseases. This network has been successful in early detection, communication and response to their priority diseases and have learned to adapt to operations in a conflict environment.

 Membership in regional networks strengthens both transboundary and national disease detection, notification and response systems and can be part of the solution to leveraging and strengthening health systems. Consider for example, PulseNet International,^9^ a network of national and regional laboratory networks committed to detecting foodborne illnesses using standardized protocols and reporting in real-time. Regional disease networks and organizations have the potential to play key roles in the development of policies and guidelines for disease detection and response. While member nations may have varied capacities and vulnerabilities, their participation in regional network(s) allows for joint advocacy across and between members and international organizations; leverages expertise, resources and capacities; and supports joint negotiation and procurement of equipment and resources for response. The Integrated Disease Surveillance and Response framework,^10^ adopted by the World Health Organization (WHO) African region, supports indicator-bases surveillance systems across its member states, providing relevant stakeholders the necessary data to improve detection and response to priority diseases at home and across the region. While we agree with the authors that including all possible organizations into an advocacy and awareness campaign can bolster recognition and validity for a regional body, we believe that applying a detailed methodology to stakeholder mapping is key to identifying organizations that have ownership and will play essential and lasting roles in regional networks, acting as champions in both advocacy and implementation.

 To this end, we suggest there is an opportunity for additional research specifically on the enablers and constraints for regional infectious disease control bodies, one that expands on infectious disease control while also continuing to take into account governance, legislative and organizational factors. Such research could be literature-based or more operational in nature, but ideally would assign metrics to assess effectiveness of networks in such a way that it could provide critical input on future design, planning and implementation (or revision of existing) networks for disease detection, surveillance and/or response. These key aspects of disease control should be approached from a One Health lens and include prevention and immunization, surveillance, laboratory, emergency response, workforce development (including field epidemiology). The development of improved measures of impact could have alignment with existing disease control frameworks operating at international scales as well. For instance, the International Health Regulations monitoring and evaluation tools (State Party Self-Assessment Annual Reporting, Joint External Evaluation) and other global health security frameworks lack clear metrics to determine whether existing and future coordination efforts, including at regional levels, have been or can be successful in addressing priority disease(s). Given these are globally applied monitoring and evaluation tools, they present an ideal opportunity for operational research on how regional networks may contribute to supporting disease prevention and control outcomes.

 To conclude, Durrance-Bagale and colleagues have presented a useful synthesis of key concepts that should be considered with respect to building successful regional networks for disease control. However, further research is needed to operationalize these findings and create more concrete tools to support future networks, as well as sustaining those already in place. We suggest that a crucial step will be to develop indicators and metrics of network success to guide this research agenda, and help establish a community of evidence-based practice in this topic area.

## Ethical issues

 Not applicable.

## Competing interests

 Authors declare that they have no competing interests.

## Authors’ contributions

 CJS and EMS collaborated in the review, preparation and submission of this commentary.
